# Peach extract induces systemic and local immune responses in an experimental food allergy model

**DOI:** 10.1038/s41598-023-28933-1

**Published:** 2023-02-02

**Authors:** H. Steigerwald, M. Krause, I. Gonzalez-Menendez, L. Quintanilla-Martinez, S. Vieths, S. Scheurer, M. Albrecht, F. Blanco-Pérez

**Affiliations:** 1 Molecular Allergology, Paul-Ehrlich-Institut (PEI), Federal Institute for Vaccines and Biomedicines, Langen, Germany; 2 Institute of Pathology and Neuropathology, Comprehensive Cancer Center, University Hospital Tübingen, Eberhard Karls University of Tübingen, Tübingen, Germany; 3 Cluster of Excellence iFIT (EXC 2180) “Image-Guided and Functionally Instructed Tumor Therapies”, Eberhard Karls University of Tübingen, Tübingen, Germany

**Keywords:** Allergy, Experimental models of disease

## Abstract

Peach allergy is among the most frequent food allergies in the Mediterranean area, often eliciting severe anaphylactic reactions in patients. Due to the risk of severe symptoms, studies in humans are limited, leading to a lack of therapeutic options. This study aimed to develop a peach allergy mouse model as a tool to better understand the pathomechanism and to allow preclinical investigations on the development of optimized strategies for immunotherapy. CBA/J mice were sensitized intraperitoneally with peach extract or PBS, using alum as adjuvant. Afterwards, extract was administered intragastrically to involve the intestinal tract. Allergen provocation was performed via intraperitoneal injection of extract, measuring drop of body temperature as main read out of anaphylaxis. The model induced allergy-related symptoms in mice, including decrease of body temperature. Antibody levels in serum and intestinal homogenates revealed a Th2 response with increased levels of mMCPT-1, peach- and Pru p 3-specific IgE, IgG1 and IgG2a as well as increased levels of IL-4 and IL-13. FACS analysis of small intestine lamina propria revealed increased amounts of T cells, neutrophils and DCs in peach allergic mice. These data suggest the successful establishment of a peach allergy mouse model, inducing systemic as well as local gastrointestinal reactions.

## Introduction

The most frequent elicitors of food allergies (FAs) in adults are allergenic plants as peanuts, wheat, fruits, nuts and soybean, together with egg, milk and fish among others^1–3^. Especially foods from the Rosacea family, including apple and/or peach are often involved in allergic reactions to plant-derived food^4^. Mainly in the Mediterranean area but also in Northern and Central Europe, non-specific lipid transfer proteins (nsLTPs) are among the most important plant allergens and are associated with severe allergic symptoms^5,6^. nsLTPs are small, heat-stable and structurally highly conserved proteins^6–8^. The sensitization to nsLTPs is dominated by the major peach allergen Pru p 3, considering peach as the primary sensitizer for nsLTP-driven clinical cross-reactivity in the Mediterranean area^6,9^. The highest amount of Pru p 3 can be found in the peach peel with a content seven times higher than in the pulp^10,11^. In line with this, peach allergy itself is described as common cause of fresh-fruit allergy^10^.

Currently, the main treatment options for peach allergy and food allergies in general are allergen avoidance or symptomatic treatment including anti-histamines as to date no cure or preventive treatment is available^12–14^. Due to this lack of treatment options, oral immunotherapy (OIT) might be considered an attractive option to induce immunological tolerance by administration of increasing dosages of allergen. However, the currently available data on OIT for peach allergy are not sufficient to recommend OIT to patients in clinical practice^15^. This shows the urgent need for further investigation to examine the molecular mechanisms underlying different types of FAs and consequently develop possible new therapies. Therefore, mouse models are commonly used to mimic and study FAs associated with several allergens such as peanut^16^, cow’s milk^17^ or tree nuts^18^. These allow the investigation of immune responses and allergic pathology without endangering health of allergic patients. In the case of peach allergy, Rodriguez et al*.*, developed a Pru p 3-allergy mouse model using an intranasal sensitization of Pru p 3 in combination with LPS and intraperitoneal challenge, to induce Pru p 3 allergy-related symptoms^19^. This model provides a good tool for the investigation of peach allergy mechanisms, triggering strong clinical signs as reduction of body temperature after provocation. However, it focusses on a single allergen and does not induce local intestinal immune responses as the gut was not involved in the sensitization process.

In this study we aimed to develop a mouse model of severe peach allergy that involves not only systemic, but also local gut-related reactions characteristic for FA. The allergic response after provocation was characterized via in vivo and in vitro analysis, considering the humoral as well as the local immune response of the gastrointestinal tract (GIT).

## Materials and methods

### Peach peel extract

Ripe peaches (Royal summer^®^ variety) were purchased from a grocery store. Freshly prepared peel was immediately frozen in acetone and dry ice and stored at − 80 °C until use. The frozen peel was ground to a powder and subsequently lyophilized. In the following, 1 g of dried peach peel powder was mixed with 10 ml of ammonium bicarbonate buffer (0.05 M), containing 2% polyvinylpolypyrrolidone (PVPP), 2 mM ethylenediaminetetraacetic acid (EDTA) and 10 mM sodium diethyldithiocarbamate (DIECA) at pH 8.3 according to the method of Björkstén et al.^20^ with slight modifications. After incubation for 2 h at 4 °C under continuous shaking, CaCl_2_ was added to a final concentration of 100 mM and incubated overnight at 4 °C. The extract was centrifuged for 30 min at 12,000×*g*, the supernatant was filtered using 0.45 µm filter, dialyzed against distilled H_2_O (dH_2_O) and lyophilized. The lyophilized peach peel extract (PE) was reconstituted in a minimum volume of dH_2_O and protein concentration was determined using Bradford protein assay^21^. Our study complied with the guidelines of the IUCN Policy Statement on Research Involving Species at Risk of Extinction and the Convention on the Trade in Endangered Species of Wild Fauna and Flora. As the peaches used in the study were purchased in the local grocery store and were marketed for human consumption, there was no risk for an endangered species or subject to any genetic modification.

### Animals

Female CBA/J mice were purchased from Charles River Deutschland GmbH and housed under specific pathogen-free conditions in the animal facility of the Paul-Ehrlich-Institut with free access to water and food. Mice were fed with a conventional AIN93G diet. All animal experiments were performed in compliance with the German Animal Welfare Act and the protocols and study were reviewed and approved by the German animal protection law (granting authority: RP Darmstadt, Germany, approval number: F107/2005). All mice were 6–8 weeks old when the experiment started and were randomly assigned to the experimental groups. We confirm that all methods in the study were carried out and reported in compliance with the ARRIVE guidelines and regulations.

### Experimental allergy model

The mice were sensitized intraperitoneally (i.p.) with 200 µg PE protein (in 200 µl; n = 5) or PBS (200 µl; n = 3) at day 0, day 7 and day 12. Imject™ Alum (Thermo Fisher Scientific, Darmstadt, Germany) was used as adjuvant (1 mg per mouse). Starting on day 19, the animals were exposed to 500 µg PE protein or PBS by oral gavage (i.g.) every second day (in 200 µl) for a total of three times. The final provocation was done three days after the last exposure by i.p. injection of 100 µg PE protein (in 200 µl) or PBS. Core body temperature and symptom score (Table S1) were monitored up to 30 min after each oral exposure and provocation. After euthanasia using CO_2_, spleens and intestine were collected for subsequent use. Collected sera and intestinal samples were stored at − 80 °C until use.

### Preparation of intestinal tissue homogenates

Intestinal tissue (10 cm length) was taken from the jejunum, Peyer’s patches were removed and the tissue was washed with cold PBS and frozen in liquid nitrogen. The frozen tissue was minced using mortar and pistil and the obtained powder was resuspended in 300 µl of cold PBS containing 1× protease inhibitor (Merck KGaA, Darmstadt, Germany). Samples were centrifuged at 12,000×*g* for 20 min and supernatant was transferred to fresh tubes. Protein concentration was determined using BCA assay (Thermo Fisher Scientific, Darmstadt, Germany) and adjusted to 5 mg/ml.

### Measurement of T-cell cytokine production

Spleens were taken and splenocytes were isolated by manual disruption. Cells were seeded at 10^5^ cells/well in a 96-well round bottom plate and were stimulated using 10 ng/ml Phorbol-12-myristat-13-acetat (PMA) and 1 µM Ionomycin for 72 h. Supernatant was collected and cytokine levels were determined using ELISA. Briefly, 50 µl of purified IL-5 capture antibody (Clone: TRFK5; eBioscience, Frankfurt am Main, Germany) or IFNγ capture antibody (Clone: XMG1.2; eBioscience, Frankfurt am Main, Germany) were coated on microtiter plates in coating buffer (50 mM sodium carbonate buffer pH 9.6) overnight at 4 °C. After blocking with 10% FCS in PBS for 2 h at RT, 50 µl of sample were added in duplicates (dilution 1:10 for IFNγ and undiluted for IL-5) and incubated for 2 h at RT. After washing, plates were incubated with 50 µl of biotinylated anti-IFNγ (Clone: R4-6A2; eBioscience; 1:1000) or anti-IL-5 (Clone: TRFK4; eBioscience; 1:1000) detection antibody for 1 h at RT. Subsequently, 50 µl of horseradish peroxidase (HRP)-labeled streptavidin solution (BD Pharmingen; 1:2000) were added and incubated for 30 min at RT. Cytokines were detected by addition of 100 µl TMB-substrate (0.525 mM 3,3′,5,5′-tetramethylbenzidine, 0.01% H_2_O_2_ in 0.21 M potassium citrate buffer; pH 3.95) by measurement of the absorbance at 450 nm. IL-4 (R&D Systems) and IL-13 (Thermo Fisher Scientific, Darmstadt, Germany) were determined by ELISA with commercial reagent kits following the manufacturer’s instructions.

### Determination of antibody responses and mMCPT-1

For determination of antigen-specific antibodies, 50 µl of natural Pru p 3 (nPru p 3; purified from peach peel as described previously^22^) or PE were coated on microtiter plates (5 and 50 µg/ml, respectively; in coating buffer) overnight at 4 °C. After blocking with 10% FCS in PBS for 2 h at RT, 50 µl of serum or intestinal homogenates were incubated for 2 h at RT. Biotinylated anti-mouse IgE (R35-118; BD Biosciences; 1:1000) antibody was incubated for 1 h at RT, followed by 30 min incubation of HRP-labeled streptavidin. For detection of antigen-specific IgG1 (sIgG1) and sIgG2a, HRP-conjugated goat anti-mouse IgG1 (Thermo Fisher Scientific; 1:2000) or rabbit anti-mouse IgG2a (Thermo Fisher Scientific; 1:2000) were used. Antigen-specific antibodies were detected by addition of TMB-substrate followed by measurement of the absorbance at 450 nm. Detection of total IgE, total IgG, total IgA as well as mMCPT-1 was performed using commercial ELISA kits according to the manufacturer’s instruction (Thermo Fisher Scientific, Darmstadt, Germany).

### Lamina propria dissociation

The lamina propria dissociation was performed following an adapted protocol from Weigmann et al.^23^. Briefly, small intestines were harvested, fat tissue and Peyer’s patches were removed. The intestines were washed with cold PBS, opened longitudinally and cut in 1 cm pieces. The samples were further washed in 1 × Hank’s Balanced Salt Solution (HBSS) containing 5 mM dithiothreitol (DTT) at 37 °C for 20 min. Intestine pieces were subsequently passed through a 100 µm cell strainer and incubated in pre-digestion solution (1× HBSS containing 5 mM EDTA and 10 mM HEPES) for 20 min at 37 °C using slow rotation. The samples were again passed through a 100 µm cell strainer followed by repeated incubation in pre-digestion solution. Afterwards, the intestine pieces were washed using 1× HBSS containing 10 mM HEPES, passed through a cell strainer and incubated in digestion solution (0.5 mg/ml Collagenase D; 0.5 mg/ml DNase I; 1 mg/ml Dispase II in PBS) for 20 min at 37 °C using slow rotation. The samples were subsequently passed through a 40 µm cell strainer and the flow through was collected in cold FCS. The isolated cells were repeatedly washed in cold PBS, counted and used for further analysis by FACS.

### FACS analysis of lamina propria cells

Single cell suspensions of lamina propria cells underwent Fc block with anti-CD16/32 (Clone: 93*;* eBioscience, Frankfurt am Main, Germany) followed by staining with extracellular antibodies (Table S2), viability dye (Thermo Fisher Scientific, Darmstadt, Germany) and if applicable nuclear staining (true-nuclear, BioLegend). For antibodies, staining panels and gating strategies refer to Table S2 and Figs. S1 and S2 respectively. Data were acquired using FACS Symphony (BD Biosciences, Heidelberg, Germany) and analyzed via FlowJo (version 10.0.8r1; BD Biosciences).

### SDS-PAGE and immunoblotting

Proteins of PE and nPru p 3 (10 µg/lane; length: 0.5 cm; thickness: 1.5 mm) were separated by SDS-PAGE under reducing as well as non-reducing conditions using 16% acrylamide gel^24^ and visualized using GelCode blue Stain Reagent (Thermo Fisher Scientific, Darmstadt, Germany). For inhibition blot, nPru p 3 and/or PE were subjected to SDS-PAGE (50 ng/cm; lange length: 1.2 cm; thickness; 1.0 mm) under non-reducing conditions and transferred to a nitrocellulose membrane by semi-dry blotting^25^, followed by blocking with TBS buffer containing 0.3% Tween 20. The membrane was incubated with Pru av 3-reactive (cherry nsLTP) rabbit serum (CE-Immundiagnostika) and BSA or nPru p 3 were added in decreasing concentrations (1, 0.1 and 0.01 µg/ml). Non-reactive rabbit pre-immune serum was used as control. To detect bound IgG, HRP-labelled goat anti-rabbit IgG (Cell Signaling) and enhanced chemiluminescence (ECL) (Merck KGaA, Darmstadt, Germany) were used. To examine reactivity of mouse serum, nPru p 3 and/or PE were subjected to SDS-PAGE (10 µg/cm; lange length: 1.2 cm; thickness: 1.0 mm) under non-reducing conditions and transferred to a nitrocellulose membrane by semi-dry blotting^25^, followed by blocking with TBS buffer containing 0.3% Tween 20. The membrane was incubated with serum pools of mice treated with PE or PBS (basal and final time points; 1:1000 diluted in TBS with 0.05% Tween and 0.1% BSA) for 2 h at RT. After washing, AP-labelled rat anti-mouse IgG1 antibody (BD Pharmingen; 1:1000) was incubated for 1.5 h at RT. To detect bound IgG, NBT/BCIP solution was added until the protein bands were visible. Reaction was stopped by addition of dH_2_O.

### Statistical analysis

The results are shown as combined data from two separate mouse experiments conducted under the same experimental settings. The results are represented as means ± SEM, and the data were statistically evaluated by Mann–Whitney U test or ANOVA (α = 0.05). The statistical software was Graph Pad Prism version 9.2.0.

## Results

### Sensitization with PE successfully induced allergy-related clinical signs in mice

**Figure 1:**
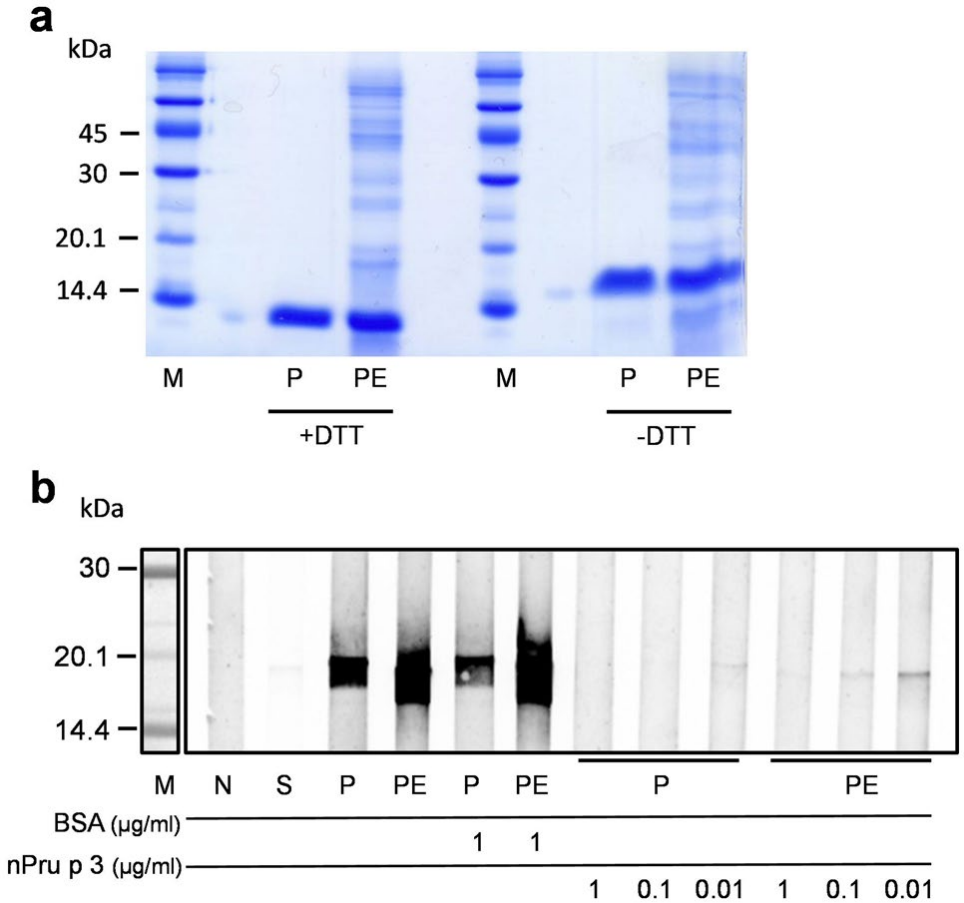
Characteristics of peach peel extract (PE). (**a**) Protein pattern of peach peel extract (PE) was determined via SDS-PAGE and Coomassie staining, natural Pru p 3 (nPru p 3; P) was used as reference; (**b**) nPru p 3 or PE were subjected to SDS-PAGE followed by immunoblot analysis. To determine allergen reactivity, nsLTP-reactive rabbit serum was used and inhibition of antibody binding was performed with increasing dosages of nPru p 3 or BSA as control. Negative control (N) or secondary antibody control (S) were incubated with either buffer or only secondary antibody.

Prior the development of a peach allergy mouse model, PE was characterized in terms of the protein pattern and antibody reactivity (Fig. 1). The PE showed a broad range of proteins with a Pru p 3 content that could be assumed to be about 50% of the included proteins (Fig. 1a). Identity of Pru p 3 in PE was corroborated by immunoblot and competition assay using nPru p 3 as inhibitor, showing that the predominant protein in the used PE was Pru p 3 (Fig. 1b).

**Figure 2:**
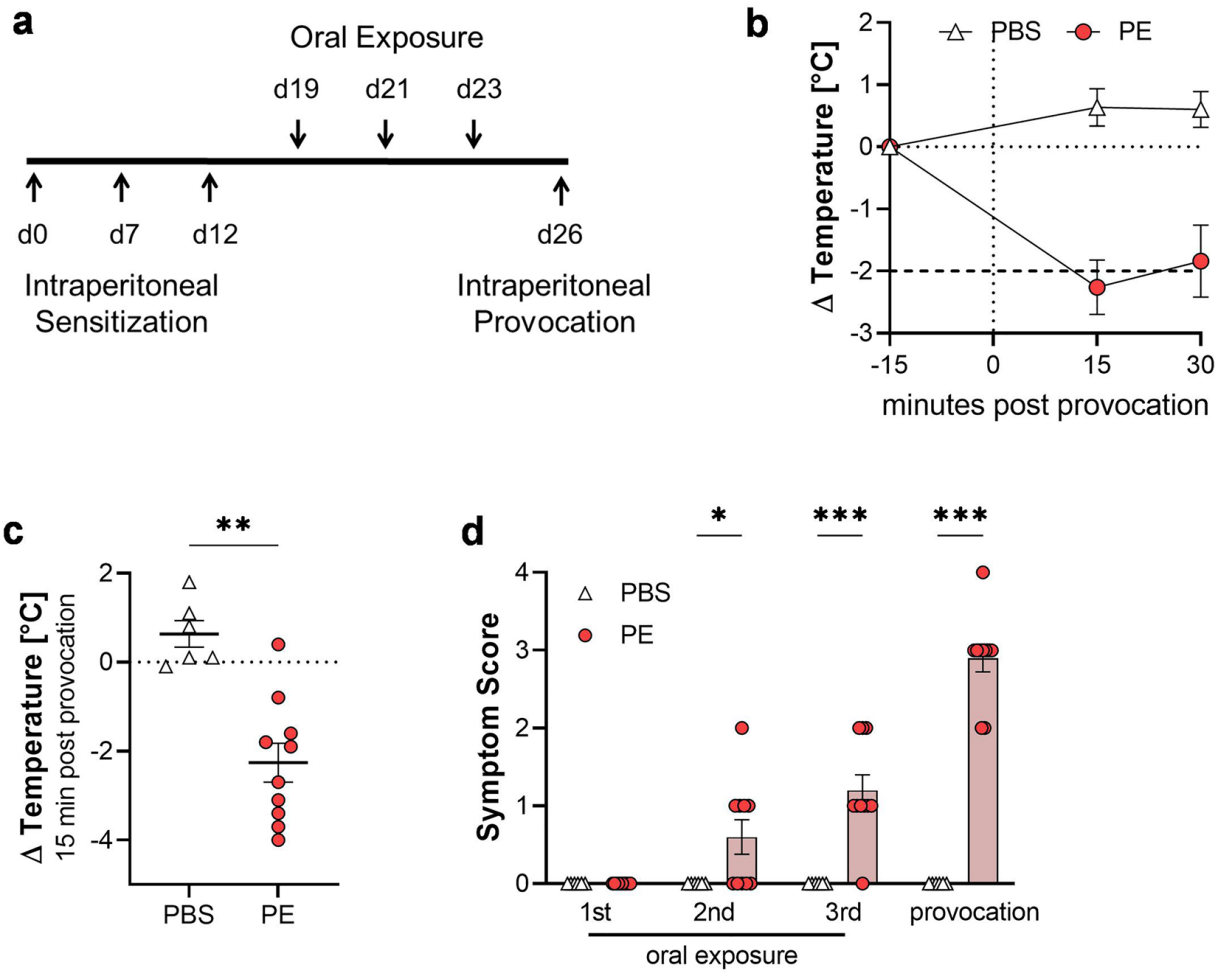
Induction of allergic signs of experimental food allergy in the peach allergy mouse model. (**a**) Schematic representation of the used model. (**b**) Body temperature was measured before (-15 min; baseline) and up to 30 min after i.p.-provocation. (**c**) Body temperature of individual mice 15 min after i.p.-provocation and (**d**) symptom score after oral exposure on third week and provocation. n = 6–10, data presented as combination of 2 experiments performed under identical settings; *p < 0.05; **p < 0.01; ***p < 0.001.

For induction of peach allergy, a mouse model including systemic and gastrointestinal exposure to peach proteins was established. Mice were treated with PE or PBS with alum as adjuvant, following the schedule shown in Fig. 2a. The core body temperature was monitored after each oral exposure (Fig. S3) and after provocation (Fig. 2b). The provocation with PE showed a significant temperature drop of more than 2 °C, which was not observed in the control group (Fig. 2b,c). As a temperature drop of more than 2 °C was defined as humane endpoint, the mice were sacrificed for animal-welfare reasons. Additionally, PE-administered mice developed allergy-related clinical signs, such as changes of behavior, consistency of the stool and ruffled fur (Fig. 2d; Table S1), which gradually increased with each oral exposure to PE when compared to the PBS-treated group. However, as expected the highest score was reached after the final provocation with PE.

### The peach allergy model is characterized by a systemic Pru p 3-specific type 2 immune response

**Figure 3:**
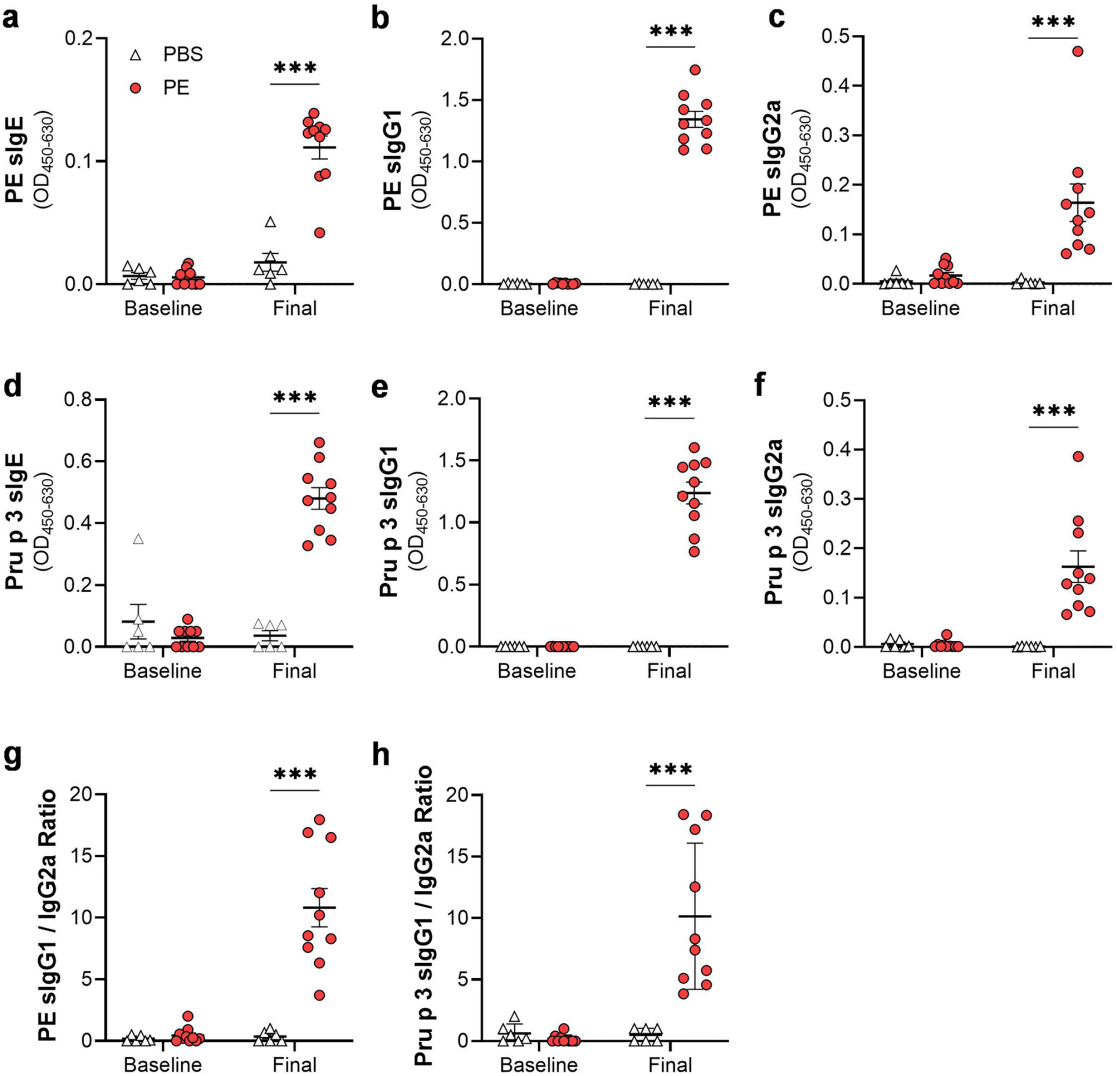
Peach extract- and Pru p 3-specific antibody response in the serum. Levels of Peach extract (PE)-specific (**a**) IgE, (**b**) IgG1 and (**c**) IgG2a, as well as Pru p 3-specific (**d**) IgE, (**e**) IgG1 and (**f**) IgG2a were analyzed in the serum of the mice before (baseline) or after (final) sensitization and provocation via ELISA. Ratio of (**g**) PE-specific and (**h**) Pru p 3-specific IgG1 to IgG2a were determined for baseline and final timepoint. n = 6–10, data presented as combination of 2 experiments performed under identical settings; ***p < 0.001.

In addition to induction of clinical signs, the humoral immune response of the mice was examined. Therefore, PE- and Pru p 3-specific antibodies were measured in the serum. Mice treated with PE developed a significant increase in PE-specific IgE, IgG1 and IgG2a (Fig. 3a–c). Comparable results were observed for Pru p 3-specific antibodies (Fig. 3d–f). The ratio of PE- as well as Pru p 3-specific IgG1 to sIgG2a was significantly increased in the PE group after provocation (Fig. 3g–h).

**Figure 4:**
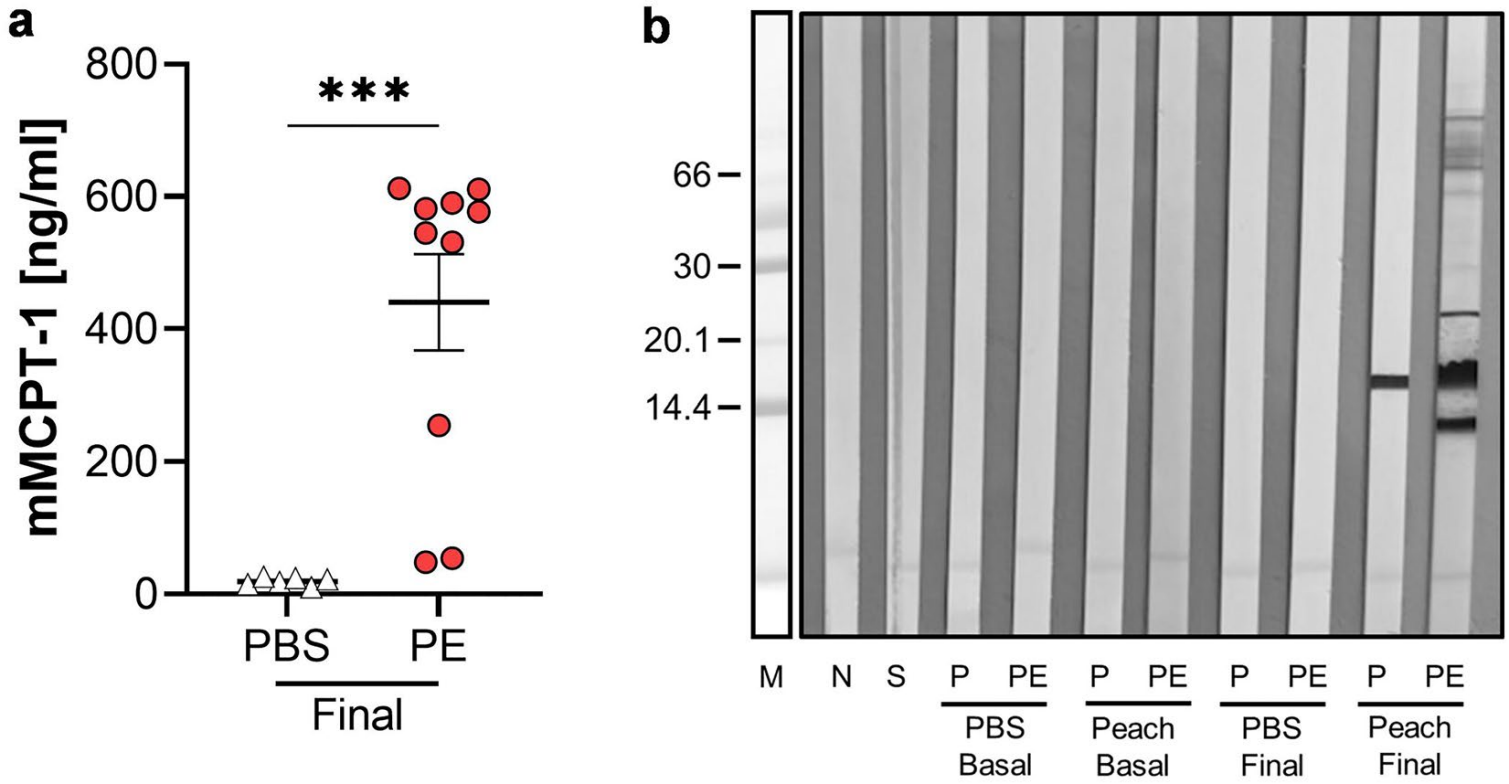
mMCPT-1 levels and IgG reactivity in serum. Levels of (**a**) mMCPT-1 were analyzed in the serum of the mice after (final) provocation via ELISA. (**b**) Peach extract (PE) and nPru p 3 (P) were subjected to SDS-PAGE followed by immunoblot analysis using serum pools from the experimental groups before (basal) or after provocation (final). n = 6–10, data presented as combination of 2 experiments performed under identical settings; ***p < 0.001.

In line with the increased levels of specific antibodies, murine mast cell protease-1 (mMCPT-1) was determined in the serum, showing a significant increase in PE-treated mice (Fig. 4a). In addition, Pru p 3 and PE specific IgG-immune response were determined by immunoblot (Fig. 4b). After provocation the PE-treated mice showed a reactivity mainly against nPru p 3 but also to other yet unidentified proteins contained in the PE.

Furthermore, total levels of immunoglobulin (Ig) were measured. PE-treated mice showed significantly increased levels of total IgE and IgG in the serum after provocation (Fig. S4a,b). In comparison, total IgA titers were reduced in the peach allergic mice after provocation, when compared to control mice and baseline values (Fig. S4c). Cytokine secretion after stimulation of splenocytes showed significantly enhanced levels of Th2-cytokines IL-4 and IL-13 after PE-treatment, whereas IL-5 and IFNγ remained unchanged (Fig. S5a–d).

### PE treatment induced local immune response in the intestine, characterized by enhanced cell infiltration and Ig production

To successfully mimic FA reaction in a mouse model, a systemic reaction but also the involvement of the intestinal tract is crucial. Here, treatment with PE showed an increase in length of both small and large intestine (Fig. S6).

**Figure 5:**
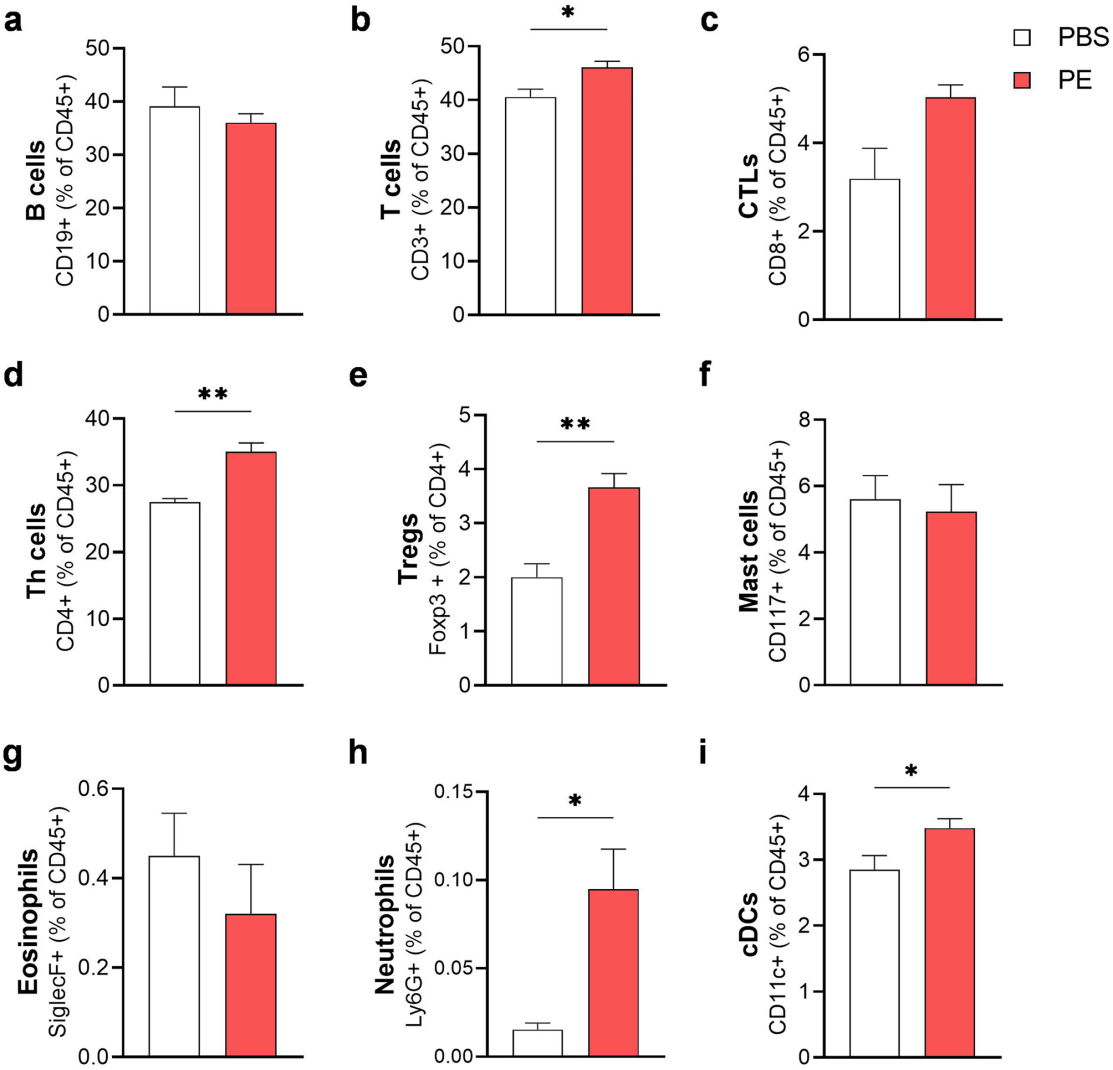
Immunological responses in the small intestine. The small intestine was enzymatically treated and isolated lamina propria immune cells were analyzed via flow cytometry. (**a**) B cells (CD45^+^ CD19^+^ cells), (**b**) T cells (CD45^+^ CD3^+^ cells), (**c**) CTLs (CD45^+^ CD3^+^ CD8^+^ cells), (**d**) Th cells (CD45^+^ CD3^+^ CD4^+^ cells), (**e**) Tregs (CD45^+^ CD3^+^ CD4^+^ Foxp3^+^), (**f**) mast cells (CD45^+^ CD117^+^), (**g**) eosinophils (CD45^+^ Siglec F^+^), (**h**) neutrophils (CD45^+^ Ly6G^+^) and (i) cDCs (CD45^+^ CD11c^+^). n = 3–5; *p < 0.05; **p < 0.01.

**Figure 6:**
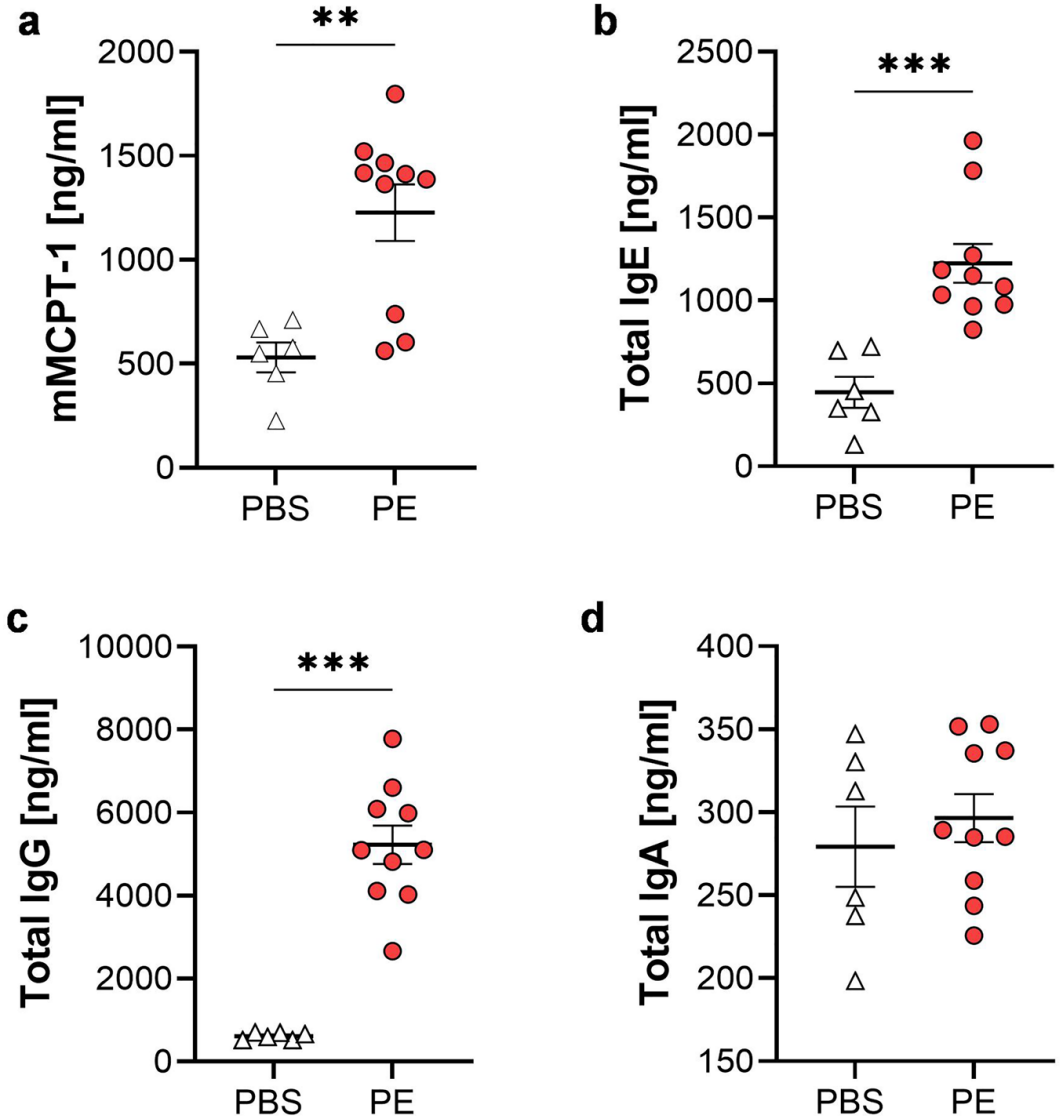
Local humoral response and mMCPT-1 in the small intestine. Levels of (**a**) mMCPT-1, (**b**) total IgE, (**c**) total IgG and (**d**) total IgA were determined in the supernatant of intestinal homogenates via ELISA. n = 6–10, data presented as combination of 2 experiments performed under identical settings; **p < 0.01; ***p < 0.001.

Analysis of immune cells from small intestine lamina propria via flow cytometry revealed induction of a local immune response in PE-treated mice (Fig. 5a–i). B cell frequency remained stable, whereas the frequency of T cells was increased in allergic mice, correlating with a slight increase of CD8^+^ (CTLs) and a strong increase of CD4^+^ (Th cells) and regulatory T cells (Tregs). Also, the frequency of neutrophils and conventional dendritic cells (cDCs) was significantly enhanced in the peach allergic mice when compared to the PBS controls. Eosinophils and mast cells were not enhanced in the PE-treated mice, however, local activation of mast cells in the intestine could be shown by significantly increased mMCPT-1 levels in supernatant of intestinal homogenates when mice were treated with PE (Fig. 6a). Further histological analysis using H&E and Giemsa staining revealed scarce neutrophils and eosinophils in the lamina propria of both treatment groups and similar numbers of mast cells present in the mucosa, sub-mucosa and muscle layer (data not shown). Measurement of local antibody concentrations in the small intestine homogenates revealed increased levels of total IgE and IgG but not IgA (Fig. 6b–d). These results suggest a substantial involvement of the intestinal system in the peach allergic mouse model.

## Discussion

Development of IgE-mediated allergy strongly correlates with dysregulation of Th1 and Th2 lymphocytes, considering especially Th2 cells as key players for both initiation and effector phase of an allergic inflammatory response^26^. Particularly in the Mediterranean area nsLTPs are frequent elicitors of FAs and sensitization to peach with its major allergen Pru p 3 is widely distributed^8,27^.

In the present study, we established a peach allergy mouse model to gain better insights in immunological reactions on peach allergy development, mechanisms and future evaluation of intervention strategies, e.g. novel therapy candidates. Different to anaphylactic reactions after exposure to food allergens in human, that cause rapid hypotension as well as skin, gastrointestinal or cardiovascular symptoms, in mice drop of the core body temperature or allergic diarrhea are typical parameters^28,29^. Mouse models artificially mimic human disease and hardly can reflect the pathology of the human conditions completely. In this study, CBA/J mice were chosen based on prior observations that LTPs as sensitization agents, induce strong and Th2-biased antigen-specific antibody responses in this mouse strain (data not shown). As oral sensitization is one of the main challenges of the establishment of a FA mouse model due to induction of tolerance or limited IgE response^30^, our results show, that combination of i.p.-sensitization, followed by oral exposure and final i.p.-provocation using PE successfully induces systemic as well as local allergy-related responses in the gut of the mice. Pru p 3- and PE-specific antibodies, including sIgE, sIgG1 and sIgG2a were significantly increased in the serum of the allergic mice, with sIgG1 being enhanced in comparison to sIgG2a, suggesting induction of a Th2-biased immune reaction^31^. This could be further confirmed by enhanced levels of Th2 cytokines IL-4 and IL-13 but not Th1 cytokine IFNγ^32^ produced by the splenocytes of PE allergic mice. In line with this, levels of total IgE, the pivotal immunoglobulin involved in type I allergy reactions^33^, as well as total levels of IgG in serum and intestinal homogenates of the peach allergic mice were strongly enhanced. In contrast, total IgA levels were significantly decreased in the serum, correlating with reports of a protective role of IgA in allergic diseases by inhibition of IgE-induced mast cell degranulation and cytokine production^34,35^.

Besides systemic effects of severe allergic reaction, the local intestinal immune response was considered of main importance for the successful establishment of a FA model. Increased numbers of T cells, especially CD4^+^ Th cells, support the hypothesis of a Th2-biased immune response. Th cells might be induced by enhanced cDCs that take up and present the allergen, leading to activation of naïve CD4^+^ cells and differentiation into Th2 cells^36^. Besides, induction of CD8^+^ CTLs has also been suggested to play a role in Th2 response to IgE-mediated FA^37^. Surprisingly, upregulation of Tregs was observed in PE-treated mice, usually known to suppress anaphylaxis in mouse FA models by controlling the responses of effector T cells and thereby inducing tolerance^38,39^. However, certain subtypes of Treg can be distinguished besides the general expression of Foxp3 and CD25^40^, which were not further characterized in this study. Likewise, the metabolic state of the identified Tregs is unknown. Tregs may be present in resting, activated or functional exhausted state, depending on the cellular and environmental stimuli that actively maintain their metabolic and immunological homeostasis^41^. Taking this together, the identified Tregs could have a different role and might not be sufficient to suppress allergic inflammation^42^. Although the number of mast cells stayed unchanged in the lamina propria of PE-treated animals, the activation status, measured as mMCPT-1 levels, was clearly increased and can be used as systemic readout for mucosal mast cell activation upon antigen-specific IgE cross-linking^43,44^. Furthermore, we did not observe an enhanced number of eosinophils in the lamina propria, correlating with comparable levels of IL-5 between the allergic and the control group. Studies showed that IL-5- and eosinophil-associated inflammation is less apparent in IgE-mediated FAs, which might explain the unchanged recruitment of eosinophils via IL-5^45,46^. More importantly, the observed increased numbers of neutrophils indicate the onset of inflammatory immune responses in the intestinal tract of the peach allergic mice, promoting attraction of DCs, T cells, monocytes and macrophages^47^. These data lead to the conclusion, that the established peach allergy model induces not only systemic but also intestinal immune responses.

Different to a previously reported experimental Pru p 3 allergy model^19^, the present model uses a combination of proteins extracted from the peach peel instead of the purified allergen as sensitization and provocation agent. In our setting, sensitization with a highly purified allergen (nPru p 3) showed low immunogenicity and was not sufficient to trigger antibody production in the mice, as observed with PE (Fig. S7a). To achieve sensitization against purified Pru p 3 it may be possible that additional adjuvant effects are needed as suggested by Rodriguez et al. by using LPS^19^.

Interestingly, in our study the levels of Pru p 3 sIgE were comparable when the mice were sensitized with PE/alum and provoked with either PE or nPru p 3 (Fig. S7b). However, levels of total IgE and IgG were higher when PE was used for sensitization and provocation. Reason for this might be a stronger sensitization capacity of PE in comparison to Pru p 3 alone due to exposure and sensitization to several allergens. As observed by immunoblot, sera of allergic mice reacted against several proteins in the extract. In regard to the MW of the detected reactive proteins, one might speculate that Pru p 7 or Pru p 1 could be included in the extract, both mainly located in peach peel and leading to severe symptoms in allergic individuals^48,49^. Furthmore, Pru p 3 and its natural ligand might be present as a complex in the peach extract, leading to enhanced allergenicity due to adjuvant-like effects of the LTP-ligand^50^. In addition, we speculate the importance of unknown potential water-soluble matrix components which mediate an adjuvant effect. Matrix effects of the PE, including fats, carbohydrates or other proteins among the allergens, might enhance allergenicity, mainly by affecting antigen availability and digestibility, enhancing sensitization capacity of the peach allergens^51^.

Preclinical studies using respective mouse models are of particular importance for allergens which could elicit severe and life-threatening reactions, making clinical studies less feasible. Thus, this model can be used to study not only peach allergy but also Pru p 3 sensitization.

## Supplementary Information

Supplementary Information.

## Data Availability

The datasets generated and/or analyzed during the current study are available from the corresponding author on reasonable request.
