# Supplementary material for: Peach extract induces systemic and local immune responses in an experimental food allergy model
Source: Sci Rep. 2023 Feb 2;13:1892. doi: 10.1038/s41598-023-28933-1 (PMC9894845; doi:10.1038/s41598-023-28933-1)
Supplement: Supplementary file 1 — Supplementary Information. [file 41598_2023_28933_MOESM1_ESM.pdf]

Supplemental Material

Peach extract induces systemic and local immune responses in an experimental food allergy model

H. Steigerwald[1], M. Krause[1], I. Gonzalez-Menendez[2,3], L. Quintanilla-Martinez[2,3], S. Vieths[1], S. Scheurer[1], M. Albrecht[1†], F. Blanco-Pérez[1†]*

[1] Vice President Research Group “Molecular Allergology”, Paul-Ehrlich-Institut (PEI), Federal Institute for Vaccines and Biomedicines, Langen, Germany

[2]Institute of Pathology and Neuropathology, Comprehensive Cancer Center, University Hospital Tübingen, Eberhard Karls University of Tübingen, Tübingen, Germany

[3]Cluster of Excellence iFIT (EXC 2180) “Image-Guided and Functionally Instructed Tumor Therapies”, Eberhard Karls University of Tübingen, Tübingen, Germany

Symptom Score	0	1	2
**Behaviour**	normal	calm	apathetic
**Fur**	normal	slightly ruffled	ruffled
**Stool**	normal	Soft	with mucus
**Body temp.**	$\Delta < -1°C$	$\Delta < -1$ to $-2°C$	$\Delta > -2°C$

Table S1: Score sheet

Score sheet parameters for quantification of clinical signs after oral exposition and provocation. Four different clinical signs were evaluated and scored from 0 to 2, with a maximum score of 8.

Target	Label	Clone	Company
CD45	FITC	30-F11	BioLegend
CD11c	BV605	N418	BioLegend
CD11b	BV421	M1/70	BioLegend
MHCII	BV510	M5/114.15.2	BioLegend
CD64	PECy7	X54-5/7.1	BioLegend
CD117	BV711	2B8	BioLegend
Ly6G	Alexa647	1A8	BioLegend
SiglecF	PE	S17007L	BioLegend
CD3	BV421	17A2	BioLegend
CD4	PECy7	GK1.5	BioLegend
CD8a	BF510	53-6.7	BioLegend
CD19	PE-BV605	6D5	BioLegend
CD25	BV711	PC61	BioLegend
FoxP3	PE	150D	BioLegend

Table S2: Antibodies used for flow cytometry

Used antibodies and specifications for flow cytometry.

T- and B-cell Panel:

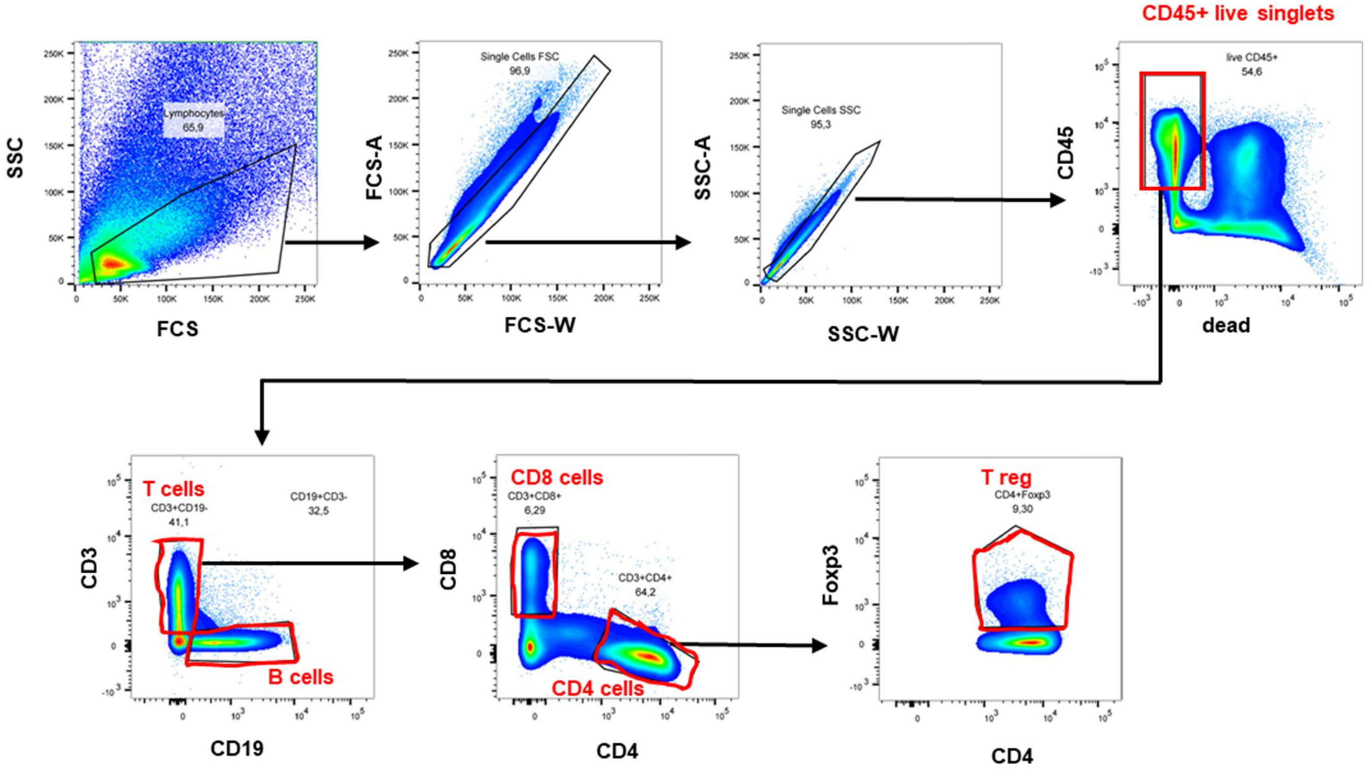

Figure S1: Gating strategy for T and B cells panel

Live lymphocytes among lamina propria cells were gated for FSC, SSC characteristics, cleaned up from doublets in FCS and SSC channel and identified as CD45+ live population (CD45+ live singlets). Among these, B cells were CD19+CD3- and T cells CD19-CD3+ populations. T cells were further discriminated for CD8 or CD4 expression. T regs were identified as Foxp3+ population among CD4 cells.

Granulocyte Panel:

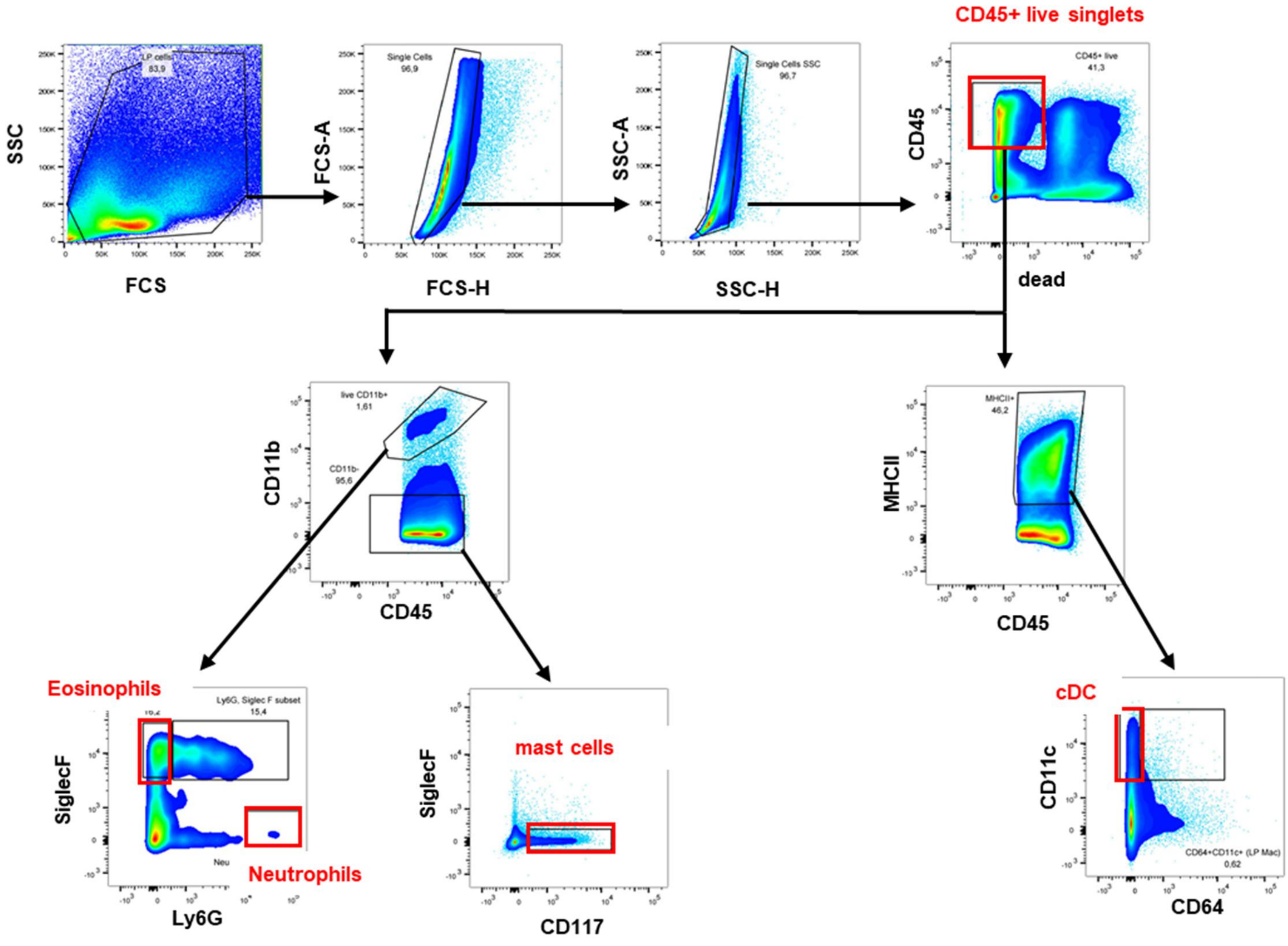

Figure S2: Gating strategy for granulocyte panel

Live lamina propria cells were gated for FSC, SSC characteristics, cleaned up from doublets in FCS and SSC channel and identified as CD45+ live population (CD45+ live singlets). Within this population CD11b+ and CD11b- populations were identified. Among CD11b+ population Eosinophils were SiglecF+Ly6G- and Neutrophils SiglecF-Ly6G+. Mast cells were identified as CD117+SiglecF- among CD11b- population. In addition, MHCII+ cells were identified among CD45+live singlets and subsequently analyzed for CD11c+CD64- conventional dendritic cells (cDC).

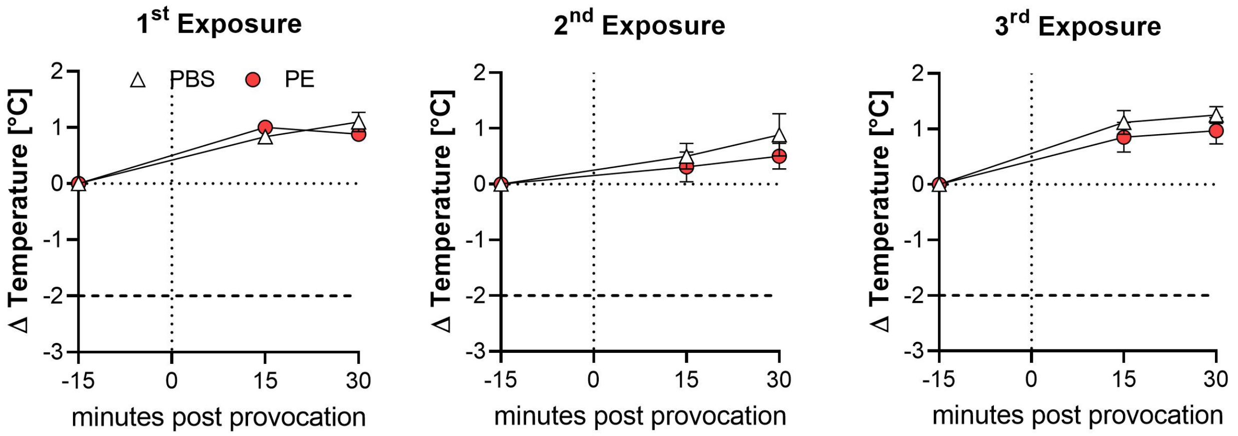

Figure S3: Core body temperature after oral exposure

Body temperature of mice after 1st, 2nd and 3rd oral exposure. n = 6-10, data presented as combination of 2 experiments performed under identical settings.

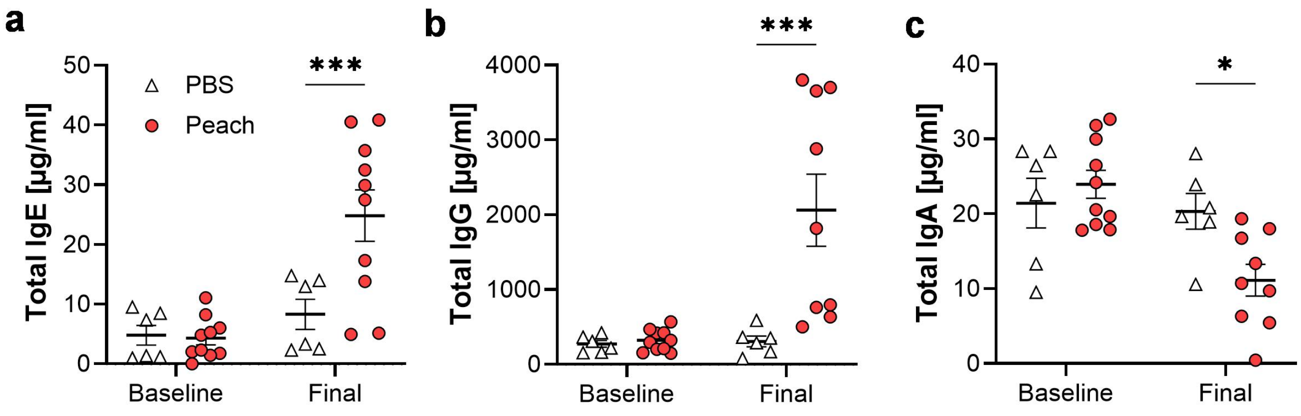

Figure S4: Total immunoglobulin levels in serum

Levels of (a) total IgE, (b) total IgG and (c) total IgA were analyzed in the serum of the mice before (baseline) or after (final) sensitization and provocation via ELISA. n = 6-10, data presented as combination of 2 experiments performed under identical settings; *p < 0.05; ***p < 0.001

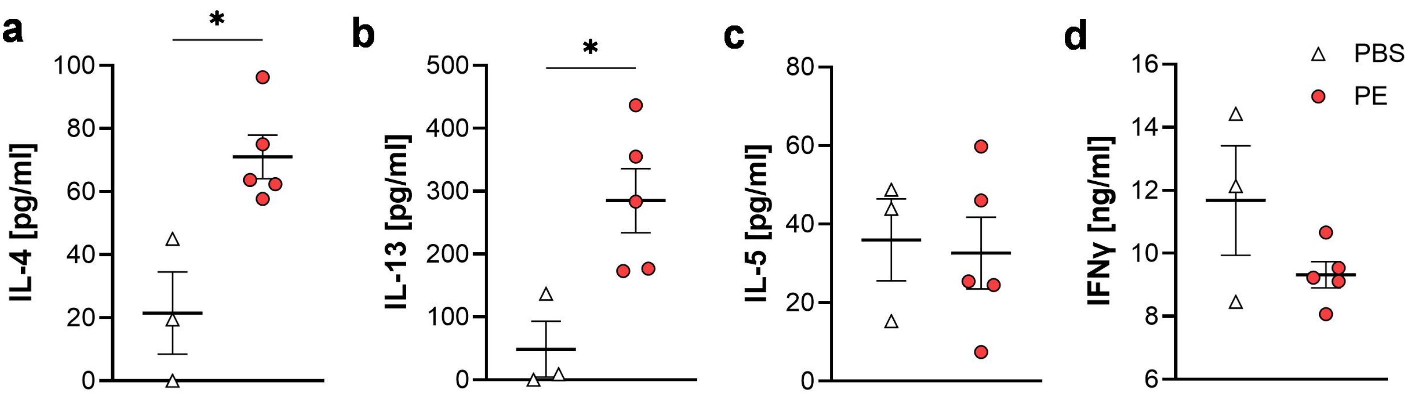

Figure S5: Measurement of cytokine secretion

Data show levels of (a) IL-4, (b) IL-13, (c) IL-5 and (d) IFNγ measured by ELISA in the supernatant of stimulated splenocytes from PBS- or PE-treated mice. n = 3-5; *$p < 0.05$

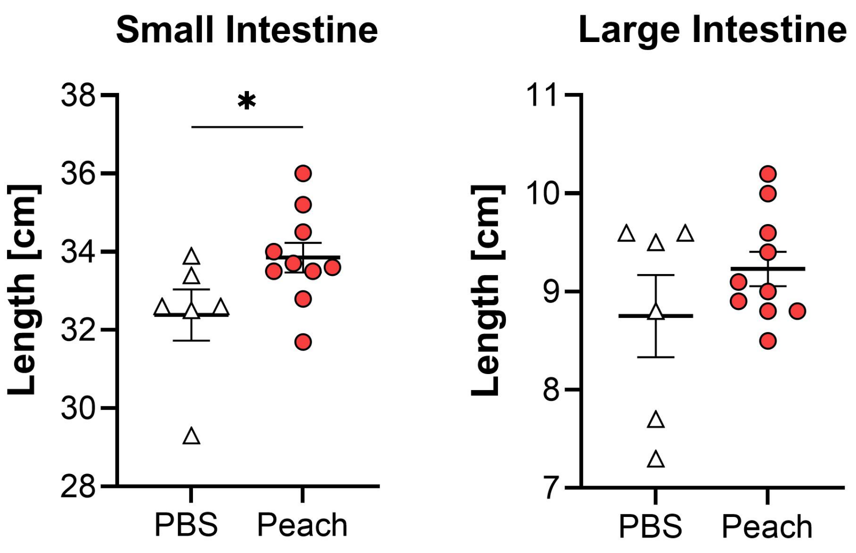

Figure S6: Measurement of intestine length

Length of small intestine and large intestine were measured after provocation and euthanasia of the mice. n = 6-10, data presented as combination of 2 experiments performed under identical settings. n = 6-10; *$p < 0.05$

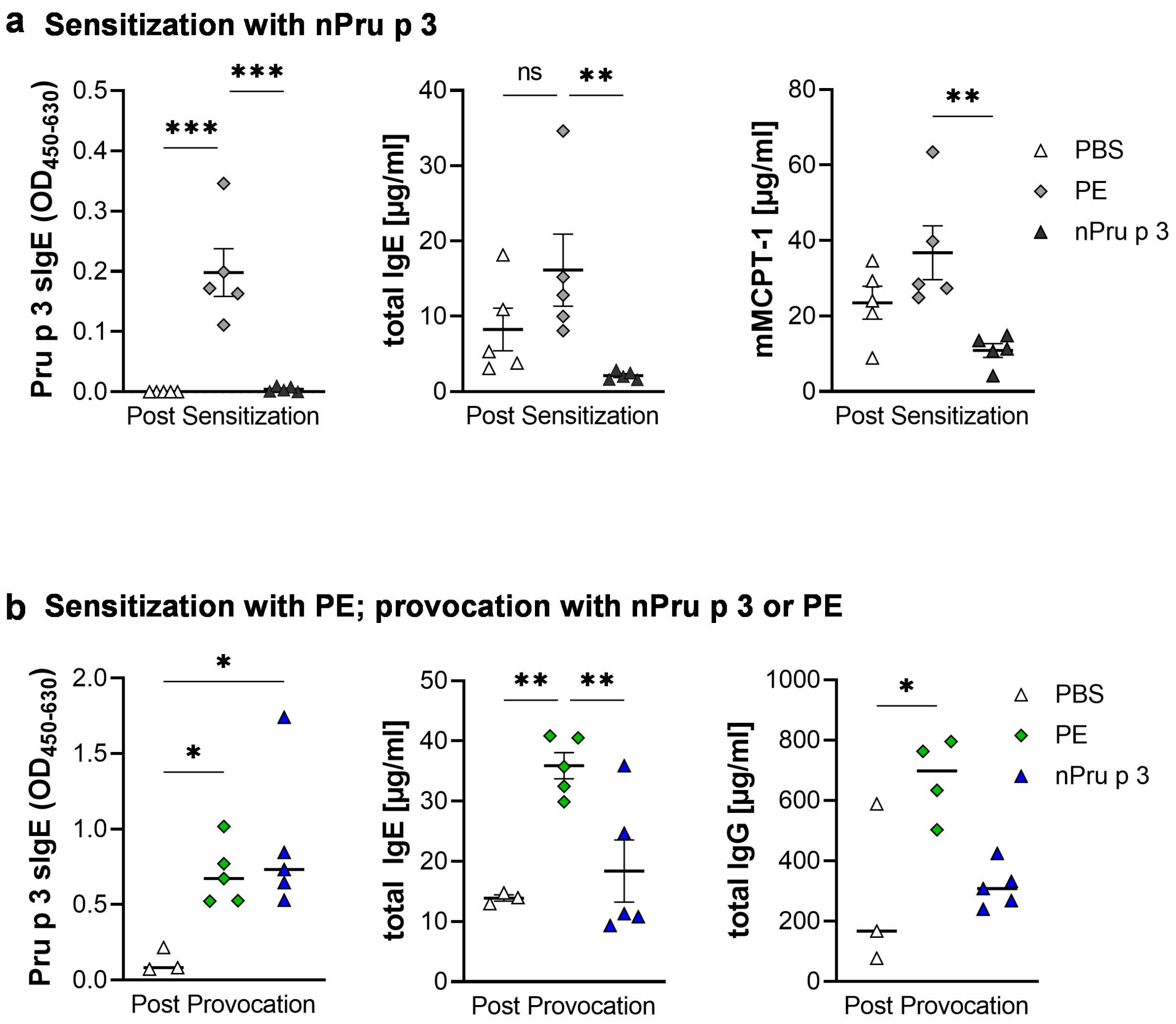

Figure S7: Antibody levels after sensitization or provocation with nPru p 3 compared to PE

(a) Mice were sensitized with 20 µg nPru p 3, 200 µg PE or PBS by i.p.-injection. Pru p 3-specific IgE (sIgE), total IgE and mMCPT-1 levels were determined in the serum of the mice post sensitization. (b) Mice were sensitized with 200 µg PE or PBS and challenged with either PBS, PE or nPru p 3. Pru p 3-sIgE, total IgE and total IgG were determined in the serum post challenge. n = 3-5; *$p < 0.05$; **$p < 0.01$.

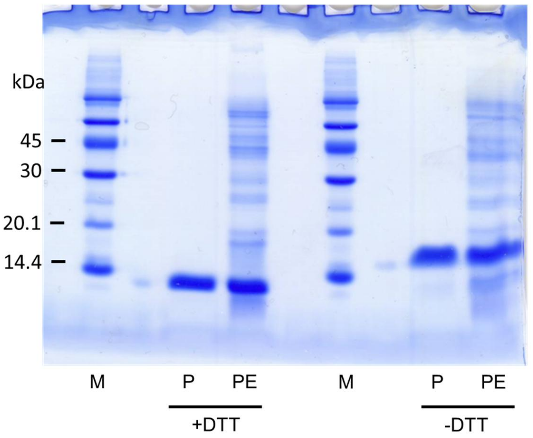

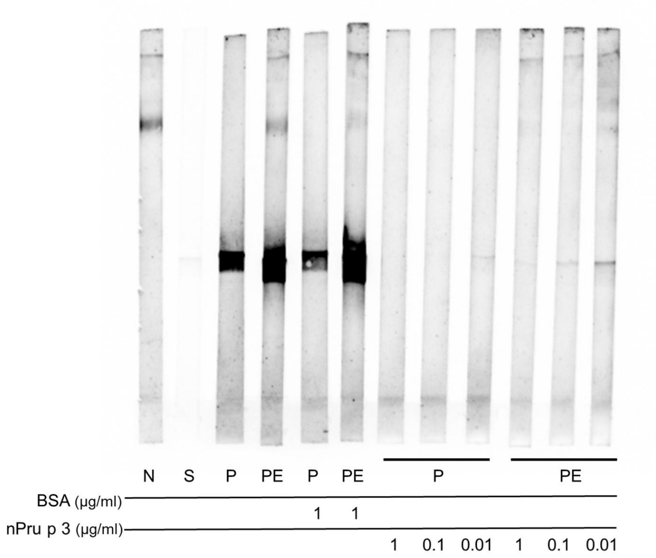

Uncut Figure 1 in compliance with Nature digital image and integrity policies.

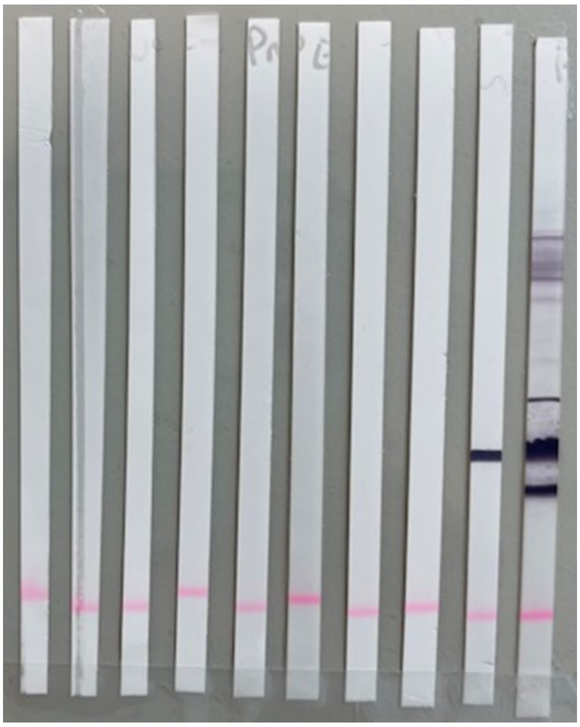

Uncut Figure 4 in compliance with Nature digital image and integrity policies.
